# Individual music therapy for managing neuropsychiatric symptoms for people with dementia and their carers: a cluster randomised controlled feasibility study

**DOI:** 10.1186/s12877-015-0082-4

**Published:** 2015-07-18

**Authors:** Ming Hung Hsu, Rosamund Flowerdew, Michael Parker, Jörg Fachner, Helen Odell-Miller

**Affiliations:** Methodist Homes (MHA), Derby, UK; Department of Music and Performing Arts, Anglia Ruskin University, Cambridge, UK; Postgraduate Medical Institute, Anglia Ruskin University, Chelmsford, UK

**Keywords:** Music therapy, Caregiving, Neuropsychiatric symptoms, Dementia

## Abstract

**Background:**

Previous research highlights the importance of staff involvement in psychosocial interventions targeting neuropsychiatric symptoms of dementia. Music therapy has shown potential effects, but it is not clear how this intervention can be programmed to involve care staff within the delivery of patients’ care. This study reports initial feasibility and outcomes from a five month music therapy programme including weekly individual active music therapy for people with dementia and weekly post-therapy video presentations for their carers in care homes.

**Methods:**

17 care home residents and 10 care staff were randomised to the music therapy intervention group or standard care control group. The cluster randomised, controlled trial included baseline, 3-month, 5-month and post-intervention 7-month measures of residents’ symptoms and well-being. Carer-resident interactions were also assessed. Feasibility was based on carers’ feedback through semi-structured interviews, programme evaluations and track records of the study.

**Results:**

The music therapy programme appeared to be a practicable and acceptable intervention for care home residents and staff in managing dementia symptoms. Recruitment and retention data indicated feasibility but also challenges. Preliminary outcomes indicated differences in symptoms (13.42, 95 % CI: [4.78 to 22.07; *p* = 0.006]) and in levels of wellbeing (−0.74, 95 % CI: [−1.15 to −0.33; *p* = 0.003]) between the two groups, indicating that residents receiving music therapy improved. Staff in the intervention group reported enhanced caregiving techniques as a result of the programme.

**Conclusion:**

The data supports the value of developing a music therapy programme involving weekly active individual music therapy sessions and music therapist-carer communication. The intervention is feasible with modifications in a more rigorous evaluation of a larger sample size.

**Trial registration:**

Clinicaltrials.gov, number NCT01744600.

## Background

In 2013, approximately 44.4 million people were reported to be living with dementia worldwide [1]. This is estimated to increase to 75.6 million in 2030, and 135.5 million in 2050. This results in an increased demand for long-term care in which effective management of symptoms is a major issue. Neuropsychiatric symptoms of dementia such as agitation, depression, apathy and anxiety are reported to affect approximately 80 % of people with dementia living in care homes [2, 3]. These symptoms are chronically present and in need of long-term support to reduce the impact on care home residents’ quality of life and caregiver stress [4].

Many current approaches in dementia care regard behaviours such as agitation as a reflection of underlying unmet psychosocial needs [5], and an attempt to communicate such needs [6]. Kitwood’s work in particular has placed importance on the quality of relationships with caregivers as key to well-being [7]. His concept of ‘person-centred care’ initiated a shift away from the more medical approach and emphasised the importance of considering the individual and their unique psychosocial needs.

In the past, psychotropic medications have been inappropriately prescribed to combat the neuropsychiatric symptoms of dementia. However, reviews indicate no or moderate effects on symptoms [8, 9] and no effect on quality of life [10]. Additionally, there has recently been growing concern regarding the over-use and risk of such medications [11, 12]. The National Dementia Strategy [13] has prompted the reduction of medication nationwide within UK care settings and highlights the importance of non-pharmacological interventions. A report commissioned by the Department of Health [14] emphasised a need for further research investigating the clinical and cost effectiveness of non-pharmacological methods.

In line with this, music therapy, a psychosocial intervention, may help to achieve the objectives of the National Dementia Strategy [13]. This therapy emphasises the development of an interpersonal relationship between the client and therapist. Active music-making forms the basis for communication in this relationship [15]. A therapy session can be seen as similar to a psychotherapy session with ensured confidentiality. However, the key difference is that clients with difficulties in using words to communicate with the therapist are enabled to express their feelings by freely singing or playing an array of percussion instruments offered in the therapy room with the therapist.

The term ‘music therapy’, defined by the World Federation of Music Therapy [16], is a state registered health discipline in the UK. On completion of the two–year training at master’s level, graduates register with the Health and Care Professions Council [17, 18] in order to practice and obtain a legally protected title ‘music therapist’. The training involves the tradition of applying psychoanalytical, psychodynamic, developmental, humanistic theories [15] and more recently theories of neuroscience in interactive live music-making as well as verbal and non-verbal communication between the therapist and client in therapy. Typical aims of the sessions for people with dementia can vary due to a person’s clinical presentation. However, for psychosocial interventions, the NICE Dementia Pathway [19] outlines the principle aims. These include providing cognitive stimulation for cognitive symptoms and managing non-cognitive symptoms such as agitation and comorbid emotional disorders such as depression and anxiety.

Literature reviews of music therapy in the field of dementia have noted its effects on symptomatic reduction [20–27]. However, although the term ‘music therapy’ is used in these reviews, some of the included studies are in fact studies of ‘music interventions’ which employ group or individualised activities incorporating listening and exercising to recorded music and singing or playing instruments along with recorded music [28–35]. These activities differentiate from the nature of music therapy as previously described and are delivered by nursing or research staff instead of a qualified music therapist. Music therapy and music interventions may deserve distinctive definitions, particularly as the interactive elements of music therapy are arguably what make this intervention unique.

Regarding active music therapy interventions delivered by a trained music therapist, this has been predominantly investigated in a group setting [21, 36–50]. Only a small number of these studies are randomised controlled trials; of this subset, there is modest evidence that group music therapy has beneficial effects on symptoms of dementia in the short-term. Reviews have raised questions over the robustness of this evidence [51], and have emphasised a need for further randomised, controlled trials with better reporting quality

With regards to the effects of active music therapy in a 1:1 setting, there is a relative paucity of studies. Recent results from Ridder [52] indicate that live interactive music therapy reduces agitation and the need for medication. Further research into this specific approach to music therapy is needed to produce more evidence supporting these findings. Furthermore, the interactive elements which make active 1:1 music therapy unique have so far been relatively unexplored [24]. More thorough investigation may yield information on the agents of change regarding how the intervention works.

Finally, there is currently no clarity on how music therapy relates to the wider context of patients’ care [27, 52]. Whether and how music therapy contributes to the multidisciplinary collaboration in delivering patients’ day-to-day care remains a less explored topic. In light of this, it is important to consider staff involvement in the provision of psychosocial interventions. The active engagement of staff and relatives and the continuing provision of tailored interventions and support are reportedly key to an intervention’s success [53]. However, this also poses further questions regarding the involvement of staff when music therapy sessions do not require staff presence and are not provided on a daily basis due to the availability of staff time and funding.

In accordance with the MRC guidance of developing and evaluating complex interventions [54, 55], the current project aims to elucidate the interactive components of individual music therapy. In addition, it aims to contribute to the knowledge gaps within this field and explore how music therapy relates to the context of care. As previous music therapy studies have not systematically involved carers as part of the intervention, the article will first illustrate the novel nature of the intervention concerning its features and process. Subsequently, the article will address issues of feasibility. These include:

1. Acceptability. Was the intervention practicable and acceptable to the care home residents and their carers? Were there any clinical concerns arising during the process?
2. Recruitment and retention. How were participants recruited? What was the recruitment rate? Did the randomisation work? What were the attrition rate and the reasons for dropouts?
3. Preliminary outcomes. What were the care staff participants’ perceptions of the intervention? Was there evidence of likely treatment effects on the measures of the resident participants’ wellbeing, symptoms and their care?

## Methods

### Trial design

This project was a feasibility study, designed as a cluster randomized controlled trial. Residents were recruited from two care homes and were randomized according to the unit they lived in within the home. In each home, one unit was randomly allocated to the intervention group, and the other to the control group. This enabled both units in each home to receive unanimous quality of standard care and input of general activities. In addition, the intervention units received weekly individual music therapy for the five month period.

A mixed methods design was employed: both qualitative and quantitative data were collected. Participants’ levels of well-being and presentation of dementia symptoms were measured through interviews and observations at Baseline, Month 3, Month 5, and as a follow-up at Month 7. Staff perceptions of music therapy were explored through semi-structured interviews at Month 6.

Information regarding the use of psychotropic medication was collected together with baseline data and demographic data at Baseline.

The study design was developed in consultation with academic and clinical professionals. Ethical approval was reviewed and granted by the National Research Ethics Service, and through Anglia Ruskin University Ethics Committees, in February 2013.

### Participants

#### Resident participants

Residents were recruited according to the following inclusion criteria:

They should:

- Reside within one of the units identified for the project
- Have a diagnosis of dementia
- Display at least two neuropsychiatric symptoms of dementia
- Be at least 40 years of age
- Display no significant health problems

Residents were recruited from the home’s pool of music therapy referrals. This pool consisted of residents referred by staff or relatives, generally due to the presence of neuropsychiatric symptoms of dementia. Residents were assessed for eligibility according to the inclusion criteria. Residents who did not meet the inclusion criteria were put on a waiting list to receive music therapy at a later date. Informed consent was obtained on behalf of all residents, through their next of kin. Participants were allocated to either the intervention or control group, depending on which unit they resided in.

Residents’ demographic and medication information was collected once recruited. Their Global Deterioration Scale scores (GDS) [56] were calculated by the researchers following discussion with residents’ keyworkers.

#### Care staff participants

Staff participants were recruited according to the following criteria:

They should:

- Work as a care assistant within one of the units identified for the project
- Have at least 3 months’ experience of working with the resident participants
- Be able to regularly work on the weekday that music therapy is provided

The research team initially sampled care staff by giving presentations on the units about the project. Staff who exhibited an interest in taking part were reviewed with regards to the eligibility criteria. Those who fulfilled the criteria and wished to take part provided consent.

### Setting

The study took place between February and September 2013 at two care homes in the UK: Home 1 and Home 2. Both cater for people with varying levels of need including residential, dementia or nursing care.

The homes were chosen according to their practical suitability for the project. Two units in each home were required to operate relatively independently of each other, in order for the control and intervention groups to be kept separate from each other. This helped prevent residents in the control group being influenced by staff perceptions and actions in the music therapy group. In both homes, the units comprised ensuite bedrooms for each resident, a communal lounge and dining area, and at least one quiet room.

In Home 1, there was accommodation for a total of 60 residents across 4 units: (15 beds and 3 staff in each); the two units used in the project were both located on the ground floor but at opposite ends of the building (15 beds and 3 carers; 15 beds and 3 carers). Home 2 provided accommodation for 68 residents across three floors: ground (20 beds, 4 carers), 1st (24 beds, 5 carers) and 2nd (24 beds, 6 carers).

### Intervention

#### Music therapy

Participants in the intervention group received 1:1 active music therapy once a week, in addition to standard care, for a period of 5 months. Each 30-minute session was conducted by one music therapist in a quiet room on the unit, and was video-recorded. Two qualified music therapists worked on the project; both had at least 2 years’ experience working in this setting and were registered with the Health and Care Professions Council (HCPC). To provide consistency and to maintain the therapeutic relationship, residents received sessions from the same music therapist throughout the project.

The sessions were based on the live interactive music therapy methods akin to the work of Odell-Miller [57] and Ridder [52]. In addition, affective neuroscience [58, 59] further informed the approach employed in the study. The therapists utilised their musical [60], vocal [61], bodily [62] and facial expressions [63]. These made up the auditory and visual inputs provided to the residents within sessions. These sensory inputs served as affective cues which could directly trigger patients’ emotional and somatic reaction.

Four key constructs of the sessions were identified. These are discussed in terms of the therapist’s auditory and visual cues:

- Auditory cues:
  1. Well-known songs were used, such as My Bonnie Lies over the Ocean. These provided repetition of musical properties, e.g. rhythm, tempo, pitch and melody, and were employed to modulate residents’ arousal. The therapists would intuitively transpose the keys of their instrumental playing and alter their singing voices to either soothe or invigorate the residents.
  2. Improvisation was also used as part of the well-known songs or vice versa to promote the residents’ participation in the joint music-making. It is necessary to note that the improvisation employed in this context did not refer to the improvisation performed in a formal classical or jazz concert. This was a process of free music-making between the resident and the therapist. It allowed the residents to respond by freely playing the instruments or simply exploring the sounds of the instruments.
  3. Talking, as similar to a psychotherapy session, also formed part of the session, allowing reminiscence and the expression of feelings. The therapists would adjust their spoken utterance and use short phrases or sentences in order to facilitate communication with the residents.
- Visual cues:
  (4) Facial and bodily expressions were given prominence and utilised as part of therapists’ musical and verbal expressions. These expressions provided non-verbal contextual cues in therapist-resident communication.

#### Video presentations

After each session, two video clips were presented to the care staff participants in the intervention group unit, in order to communicate elements of music therapy to carers. The protocol of the presentations was based on the NICE Dementia Pathway [19]. This aimed to address:

1. How neuropsychiatric symptoms were minimised.
2. The possible causes of such symptoms.
3. How the therapist made use of the participants’ remaining abilities to enhance residents’ expressions, mood and cognitive and sensorimotor functioning within sessions.

A communication sheet was used to record the main points of the presentation. Carers were encouraged to also record instances in which they had used music therapy ideas during the week with residents.

Care staff education, in particular teaching carers to adjust their interactions with people with dementia, is noted to have lasting effectiveness for managing neuropsychiatric symptoms [23]. The Video Presentation element as an intervention in this project was developed with this view to extend the effects of weekly music therapy into the staff day-to-day practice.

The video clips allowed the music therapist and research assistant to clearly demonstrate what could be done to prevent the triggers of the symptoms and to effectively minimise the symptoms when they appeared. This weekly intervention aimed to embed the practicable knowledge, methods and techniques into carers’ interaction with the residents in their everyday life.

As staff active involvement is one of the key strategies in making psychosocial interventions work [53], weekly video presentations were identified as the most time-efficient method in this study to sustain consistent staff involvement. The development of the intervention took into account the factors such as staff time, priorities and risk that could impede the implementation. For example, staff participation in the therapy sessions could put a strain on their time for fulfilling other duties. Their presence in the sessions could also be an intrusion into the resident participants’ one–to-one therapy and compromise the confidentiality. By carefully reviewing and selecting the video clips, the music therapist and research assistant were able to filter out certain sensitive information and only relay the most useful information to the staff in the 15-minute presentations.

The staff who participated in the weekly video presentations were encouraged to apply what they had learned as well as their own ideas into their daily practice. The feedback from the staff during the video presentations also in turn informed the music therapists’ working strategies in the therapy sessions. This intervention explored the possibilities of using music therapy as an on-going training tool which could be included as part of carers’ day-to-day work [64], p.1126. This would intend to help improve staff knowledge of their patients and staff confidence and skills to interact with the patients. As a result, a task-oriented care approach may be shifted to a resident-oriented approach [65].

#### Control group

The control group received standard care only for 5 months. This consisted of medical and personal care, provision of basic needs, and activities carried out as usual within the home such as chaplaincy services, entertainment and leisure activities.

### Outcome measures

The Neuropsychiatric Inventory and Dementia Care Mapping were conducted at four time points: at Baseline, Month 3, Month 5, and as a follow-up at Month 7.

#### The Neuropsychiatric Inventory for Nursing Homes (NPI-NH)

The NPI-NH is a semi-structured interview for use in nursing homes that collects data on residents’ neuropsychiatric symptoms of dementia. Interviews were conducted with care staff participants by two researchers who followed the suggested protocol [66]. The interview assesses 12 areas of behaviour and neuropsychiatric functioning: delusions, hallucinations, agitation, depression, anxiety, euphoria, apathy, disinhibition, irritability, aberrant motor behaviour, night‐time behaviour and appetite disturbance. Each symptom is scored according to its perceived frequency (scale of 0–4), severity (0–3) and level of occupational disruptiveness to staff (0–5). Occupational disruptiveness concerns the effect of the symptom on the carer’s daily practice, with regards to work routine and emotional impact, with 0 indicating no disruptiveness and 5 indicating ‘very disruptive’, A high score also indicates that this behaviour is a major source of distress for staff and other residents, which requires carers’ time usually devoted to other residents or activities.

An overall score for each symptom is calculated by multiplying frequency by severity. A score of greater than 3 is commonly taken to be indicative of clinically relevant symptoms [67]. Each score is added together to give a total NPI score; the highest achievable score is 144.

The NPI has been proven to be valid, reliable and sensitive to change, and has been used in a number of clinical trials [39, 47, 68].

#### Dementia Care Mapping (DCM)

DCM is an observational tool used within institutional settings that provides information on residents’ well-being and the quality of care delivered by staff [69]. Several studies have posited that relative wellbeing is an appropriate outcome measure in dementia care [6, 70], and DCM has been used as an outcome measure in a number of clinical trials [70, 71].

During mapping sessions, the research assistant recorded residents’ behaviours, mood, engagement and interactions with staff over a defined time period. Participants were observed for two consecutive hours over lunchtime. Residents’ behaviours, mood and engagement were systematically coded within 5-minute time frames. Behaviours were coded as a letter, eg. ‘F’ for eating, ‘N’ for sleeping, ‘L’ for engaging in a leisure activity. Mood and engagement were scored between −5 and +5, with −5 indicating very low mood and/or low level of engagement with the environment, and +5 indicating considerably positive mood and/or high level of engagement with the environment. The mood and engagement scores for each time frame were then analysed to give an overall ‘wellbeing’ score between −5 and +5, with a negative score indicating ill-being and positive score indicating well-being. In addition, staff-resident interactions were recorded as and when they occurred during a dementia care map, according to type and potential for well-being, and were named as ‘personal detractors’ or ‘personal enhancers’. The study explored the percentage of personal enhancers in carer-resident interactions contributing to the quality of person-centred-care being delivered.

#### Physiological data

Data on residents’ physiological state were collected during sessions and immediately before and after. Two devices were used: a Polar heart rate monitor and an Affectiva Q Sensor. The heart rate monitor attaches to the wrist and the chest, and measures residents’ heart rate and heart rate variability. The Q Sensor attaches to the wrist and measures levels of skin conductance, skin temperature and bodily acceleration (movement).

The devices were attached by a carer and researcher. The resident was then encouraged to relax in the therapy room in a comfortable chair for 15 min before the session. This was repeated for 15 min following the session. This aimed to provide data on residents’ physiology at a resting baseline state.

The analysis of the physiological data from the heart rate monitor and skin conductance sensor is a specialised field and will form a separate study of psychophysiology. It is hoped this will shed light on how the residents responded within the sessions.

#### Semi-structured interviews

During the 6th month, semi structured interviews with care staff were conducted to explore their perception of music therapy and its effects on residents within the intervention group. The interview questions were:

- What are the challenges you experience in your daily practice?
- Do you think music therapy can have a positive or negative effect on the residents?
- Have you seen a change in residents’ symptoms or well-being since they started receiving music therapy?
- What did you feel about the video presentations? What made the strongest impression on you?
- Has your experience of music therapy changed the way you work?
- What would you like to learn from the music therapists?

These were transcribed and themes were identified and re-analysed by two investigators who crosschecked for reliability. NVivo computerised package version 10 was used for data analysis.

### Data collection procedure

In each care home, a quiet room on the intervention unit was utilised for the music therapy sessions. A separate room was employed for the reviewing of data. At the start of the day the music therapist and research assistant set up the music therapy room with an electronic keyboard, two chairs and various percussion instruments including xylophones, a drum, suspended cymbal, tambourines and beaters. Two video cameras were set up with tripods to record the sessions, one as a back-up. In the second room the research assistant set up the computers, and set the physiological sensors to the correct time.

Before each session the resident was asked whether they were happy to attend. If yes, the physiological sensors were attached by a carer. The resident was then seated in the music therapy room for 15 min to collect baseline physiological data. The music therapist conducted the session, after which the resident remained seated for 15 min resting time. The resident was then assisted back to the communal area and the sensors were removed by a carer. The research assistant uploaded the data from the sensors and the video camera.

This was repeated with the remaining residents. The research assistant and music therapist then reviewed the video recordings of sessions and selected clips. A presentation using these clips was given to participating carers in the afternoon.

The research assistant collected data for the outcome measures at four time-points; 2 weeks were allocated at each time-point to collect this data. The research assistant carried out the 2-hour Dementia Care Map over lunchtime in each unit, and observed the participants over this period from a discreet position in the corner. The research assistant carried out the NPI-NH interviews with care staff participants before and after the Dementia Care Map, in a quiet room on the unit. This routine was repeated in the remaining units on subsequent days.

### Sample size

Being a feasibility study, a formal sample size calculation was not performed. The sample size was selected based on what would be feasible for the music therapists, researchers and care staff. Key considerations were the amount of time necessary to conduct interviews, sessions, and review video recordings. It was estimated that a typical day would consist of: 1 h for therapist and research assistant to prepare instruments, recording equipment and physiological devices; one 30-minute music therapy session per resident with 15-minute resting time pre- and post-session; 1 h per session for therapist and research assistant to review video recording and select clips; one 15-minute presentation to carers including resulting discussion and questions; and a flexible amount of time before and after each session for a carer to attach and remove the sensors. Regarding staff participants, a key consideration was the number able to participate without compromising their daily delivery of care to all residents on the unit.

In view of this it was decided that 3 sessions per day could be conducted. An optimum number of 16 resident participants and 10 staff participants was calculated.

### Randomisation

Randomisation was carried out between units (cluster randomisation) to reduce contamination across the control and intervention groups.

After participants had been recruited by the researchers, randomisation was conducted by the study statistician independently of the researchers. Random decimals were generated using the RAND() function in Microsoft Excel 2008 for Mac Version 12.3.5. These were used to allocate the care home units to either the control or intervention group. The randomisation was stratified by care home so that there was an allocation of both control and intervention represented within each care home. The outcomes were then provided to the researchers who then implemented the allocations.

### Blinding

Blinding was not carried out. It would not have been possible to conceal which group participants were in, due to the nature of the intervention. The music therapy sessions took place within the care homes and participants could be observed and heard attending the sessions.

### Statistical methods

To analyse the effects of music therapy compared to standard care, two approaches were used. First, descriptive analyses were conducted, in which means and standard deviations were analysed at each time point. The developments over the 7 months between groups were then displayed graphically.

Secondly, inferential statistical analyses were conducted using repeated measures analysis of variance (ANOVA), with the within-subjects factor Time (with levels Baseline, 3 months, 5 months, and 7 months) and between-subjects factor Condition (with levels Control and Intervention). This allowed investigation of whether there were significant differences in levels of symptoms, wellbeing, occupational disruptiveness and Personal Enhancers over time, within each group and between the two groups. The analysis provides contrasts for the Time x Condition interaction. These assessed whether the change between two time points was the same for the Control and the Intervention. This was performed for the pairs of time points Baseline vs 3 months, Baseline vs 5 months, and Baseline vs 7 months. This interaction effect was obtained as a contrast in SPSS defined by the two subcommands MMATRIX and LMATRIX. The effect size measure partial eta squared was obtained from this and was converted to Cohen’s d according to DeCoster [72]. This was a feasibility study and due to the small sample size, certain statistical requirements could not be satisfied, therefore no cluster adjustment or multiple outcomes were performed. SPSS computerised package version 20 was used for all statistical analysis.

## Results

### Recruitment

Recruitment was carried out from January to February 2013. A total of 27 participants were recruited into the study, comprising 17 residents and 10 staff from two care homes. Prior to data collection, 76 resident participants and 12 staff participants were assessed for eligibility. 17 residents (22 %) met the inclusion criteria and consent was sought from next of kin. Consent was given for all 17 resident participants (100 %) and all completed the baseline assessment. 10 staff (83 %) met the inclusion criteria and all (100 %) gave consent. After the baseline data collection in February 2013, the participating residents and carers were allocated to either standard care or music therapy according to how the unit they resided or worked in had been randomised. At Home 1, there were 4 residents and 2 carers in the intervention group, and 3 residents and 1 carer in the control group. At Home 2, 5 residents and 5 carers recruited for the intervention group, and 5 residents and 2 carers recruited for the control group. Thus in total, 9 residents and 7 carers were allocated to the music therapy group, and 8 residents and 3 carers were allocated to the standard care group. Follow-up data was collected at the end of the study in September 2013.

### Baseline data

Baseline characteristics (displayed in Table 1) were collected following recruitment of participants. The majority of resident participants were female (94 %). The age range was 56–98 years, with 84 years as the mean age.

**Table 1:** Baseline evaluation

Features at baseline	Music therapy group (*n* = 9)		Standard care group (*n* = 8)	
	*n*	Mean (SD)	*n*	Mean (SD)
Global deterioration scale	9	5.89 (1.05)	8	5.50 (1.31)
Symptom score (NPI-NH)	9	14.33 (9.85)	8	15.63 (7.87)
Wellbeing score (DCM)	8	0.85 (0.52)	8	1.54 (0.53)
Age	9	84.56 (6.64)	8	82.50 (13.04)
Female gender, %	8	89 %	8	100 %
Months lived at care home	9	20.33 (10.58)	8	19.75 (20.14)
Medication, %				
Antipsychotic medication	1	11 %	0	0 %
Antidepressant medication	3	33 %	4	50 %
Antidementia medication	3	33 %	3	38 %
Diagnosis, %				
Alzheimer’s	4	44 %	3	38 %
Vascular	2	22 %	0	0 %
Frontal lobe	2	22 %	0	0 %
Lewy Body	1	11 %	1	13 %
Mixed	0	0 %	1	13 %
Unspecified	0	0 %	3	38 %
Staff age	6	38.17 (17.11)	3	38.00 (11.53)
Staff length of employment	6	32.33 (28.90)	3	23.33 (28.31)
Staff female gender, %	5	83 %	2	67 %
Personal enhancers (DCM)^a^, %		87.50 % (17.68)		94.45 % (7.85))
Occupational disruptiveness (NPI-NH)	6	2.67 (1.61)	7	3.00 (1.49)

^a^the percentage of personal enhancers is calculated using the following formula:

((Personally enhancing interactions + 2(Highly personally enhancing interactions)) / (2(Highly personally detracting interactions) + Personally detracting interactions + Personally enhancing interactions + 2(Personally enhancing interactions))) * 100

The majority of resident participants were diagnosed with dementia of Alzheimer’s type (41 %). The remaining residents had diagnoses of Vascular, Frontal Lobe, Lewy Body and Mixed Type Dementia, while 18 % of participants had an Unspecified dementia diagnosis. All diagnoses were made in accordance with the Diagnostic and Statistical Manual of Mental Disorders (DSM-V). At the start of the project, 6 % of residents were prescribed anti-psychotic medication, 41 % were prescribed anti-depressant medication and 35 % were prescribed anti-dementia medication.

The majority of staff participants were female (78 %). Staff ranged in age from 21 to 60 years, with a mean of 38 years. The length of staff employment prior to recruitment ranged between 3 months and 7 years, with a mean duration of 2 years 5 months. One member of staff did not provide demographic data and is excluded from Table 1.

### Retention

#### Residents

3 resident participants from the music therapy group (33 %; 18 % of total number of resident participants) died due to a decline in physical health before the 3 months data collection. Their baseline results were not included in the final analysis.

**Fig. 1:**
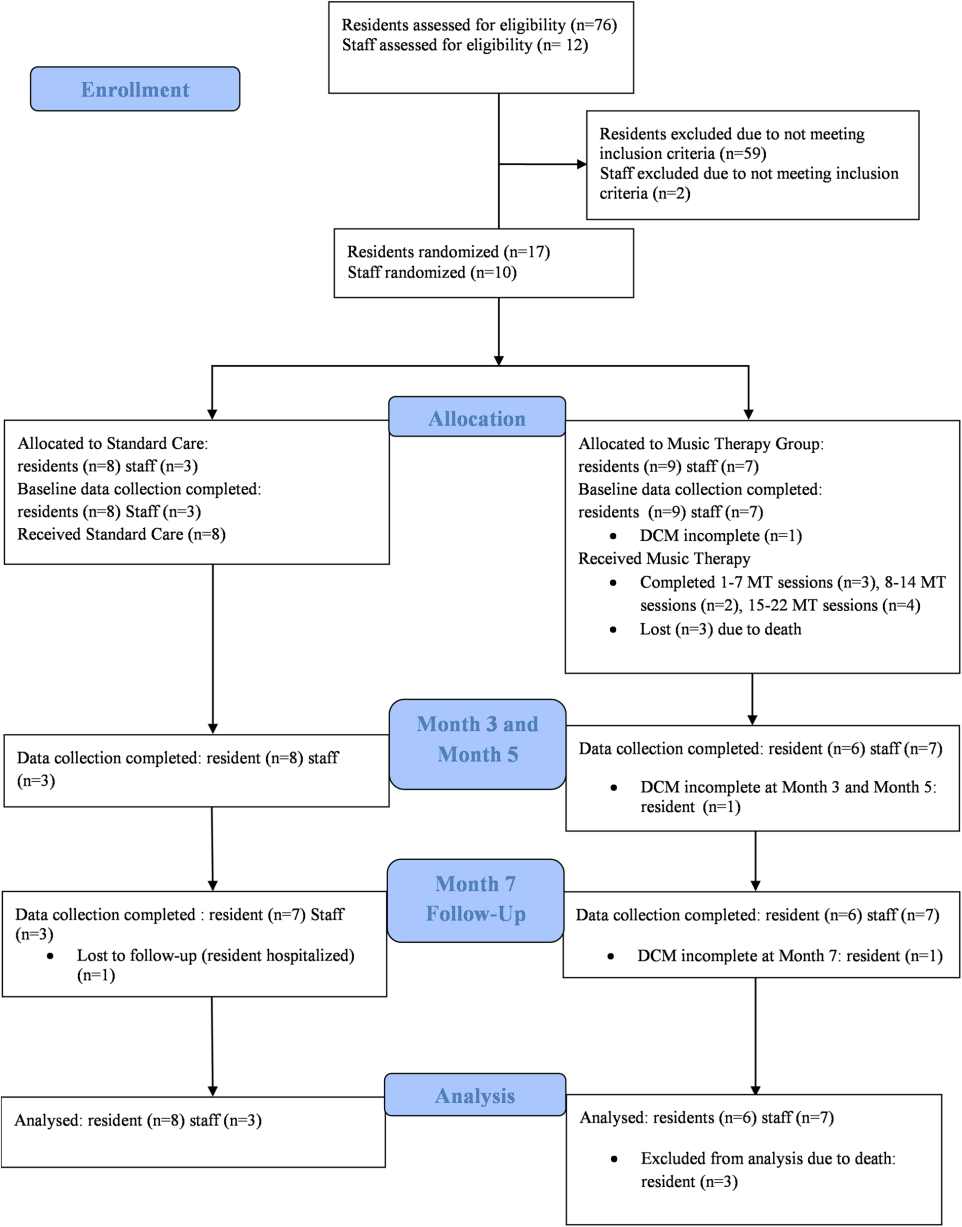
Participant flow

Missing data is due to 1 resident participant from the standard care group being hospitalised before the 7 months data collection, and 1 resident participant from the music therapy group absent during the dementia care mapping observations (together comprising 12 % of total resident participants). The results were analysed respectively using the data from the 13 completers of Neuropsychiatric Inventory (71 %) and 12 completers of Dementia Care Mapping (71 %). See flow diagram, Fig. 1, for details.

#### Staff

1 member of staff from the control group (11 % of total staff participants) dropped out of the study following recruitment, before data collection commenced.

#### Sessions

In the music therapy group, 22 weekly therapy sessions were offered to 8 residents. One resident was offered 19 as consent was not received until later in this case. The mean number of attended sessions was 15 (75.11 %) (standard deviation = 4.08). On average, 5 sessions were missed (standard deviation = 3.94).

### Numbers analysed

Only data from the completers (14 residents) were analysed. Intention to treat analysis was not performed due to the low sample size, as the effect of dilution would have been high.

### Medication

The majority of residents’ medication remained the same throughout the project. However, one resident in the music therapy group was previously prescribed an antipsychotic drug (Quetiapine) which was resumed at the beginning of the music therapy programme. This was replaced with an alternative, Risperidone, 3 weeks later. The dosage was subsequently reduced. Several weeks after the music therapy programme had finished, the dosage was increased again. In addition to the antipsychotic medication, this participant’s antidepressant medication (Citalopram) was reduced in dosage towards the end of the music therapy programme.

One participant in the standard care group commenced an antipsychotic drug (Risperidone) two months after the project started. This prescription remained for the duration of the project.

### Case example

Both the music therapy sessions and video presentations employed in the study were complex interventions involving interconnecting and interactive components as previously outlined. In order to shed light on the process of the interventions, as part of our intent to explore the feasibility, a case example qualitatively illustrating the process of a single session for B is included below. B’s next of kin gave consent for details of her therapy sessions to be published.

#### Context

B was 89 years old and had resided in the care home for the past 12 months when the music therapy project started. She had a diagnosis of dementia with Lewy Bodies and had scored 6 on the Global Deterioration Scale. Due to her condition, B displayed auditory and visual hallucination and paranoid thoughts. These could often cause a high level of agitation or anxiety for B as she felt others were talking about her and looking at her. As a result, anti-dementia, antidepressant and antipsychotic medications had been prescribed to manage B’s symptoms. B used a walking stick and could seem quite breathless whilst moving around the unit in the home. When she became agitated, she could wave her stick and hit the staff and her fellow residents. The care staff had found managing B’s symptoms extremely challenging and disruptive to their work routine.

#### Pre-therapy preparation

Before commencing a music therapy session, one of the care staff or the research assistant would help to put the heart rate and skin conductance sensors onto B. This was to collect B’s baseline physiological data and B would normally be invited to sit and rest in the quiet lounge on her own for a duration of 15 min. At the end of the baseline data collection, a music therapy session would take place in the same room whilst the sensors continued to collect the data during the session. Another 15 min were needed for data collection after each music therapy session. B would be encouraged again to sit and rest during this time.

#### Music therapy session

B had been walking around and appeared very agitated prior to arriving in the lounge. When the music therapist helped attach the sensors, B refused to wear them and displayed verbal agitation, shouting at the music therapist and demanding the therapist to stop staring at her. The therapist respected B’s decision and therefore decided to carry out the session without the sensors in place.

In view of B’s agitated behaviour, the therapist considered using arousal regulation [73] to ameliorate B’s symptoms. This could be carried out within interactions with B, utilising the therapist’s musical, vocal, bodily and facial expressions as emotional cues. The therapist initially softened her voice to greet B and explained what they could do in the session. However, the therapist’s verbal expression seemed to aggravate B’s agitation. As the verbal cues failed, the therapist then sat quietly next to her and slowly introduced a soothing melody as a musical cue on the keyboard placed in front of them. All at once, B reached her hands to the keys to initiate a short well known tune but appeared unable to complete the melodic sequence. The therapist recognised the tune and therefore completed the tune, following on from where B stopped. B now started making eye contact with the therapist and showed subtle smiles whilst listening to the tune being repeated by the therapist. Subsequently, B moved her hand in the air to the beat of the music. After a moment, she turned to the therapist and said that the tune was too slow. The therapist speeded up the tempo of the music and produced a percussive and brighter timbre. Quickening the tempo and altering the timbre seemed to produce further effects to enhance B’s mood. B nodded her head to acknowledge the therapist’s effort.

As the therapist’s playing gradually came to an end, B exhibited a pleasant smile and told the therapist ‘I liked that’. The therapist reinforced B’s positive verbal expression by humming the tune again whilst moving her body and shoulders and smiling at B. At this time, no sign of agitation could be observed. For the rest of the session, the therapist sang and played a few songs to B. Occasionally, B joined in singing and playing freely on the keyboard with the therapist. This also led to B sharing with the therapist her fond memories of learning to play the piano as a child. Towards the end of the session, B appeared good spirited whilst recalling other children coming by her window calling out B’s name and asking her to play them a tune.

#### Video presentation

After the therapy session, the music therapist alongside the research assistant reviewed the videoed session and selected 2 video clips. The clips were incorporated during the presentation to the staff which followed the protocol set up for this project. One of these clips captured the moment when B shouted at the therapist, but eventually become settled in mood as the therapist repeated playing the tune B initiated on the keyboard.

The therapist and the research assistant explained to the staff that B seemed to have had a very positive experience of learning and playing the piano in her childhood. As B retained the abilities to retrieve autobiographical memories, it allowed the piano to be an emotional visual cue which could trigger the associated memories and emotions. The staff were advised to utilise this cue when B became agitated. Engaging B to sit in front of the piano in the unit and talk about her experience of playing the piano could possibly minimise the level of agitation.

Additionally, the understanding of emotions which could be triggered by physiological changes such as heart rate, muscle tone and endocrine release [74] and the concept of Somatic Marker Hypothesis (SMH) [75] enabled the therapist to identify a possible link between B’s experience of breathlessness and her agitation. B often appeared to be breathless after walking for a while. This seemed to consequently result in her agitated behaviour as she did not seem able to cognitively appraise what might have upset her mood. The staff usually walked with B to the quiet lounge for music therapy and on many occasions she arrived in an agitated and breathless state. The therapist advised the staff to observe B when she walked around the unit and to be vigilant to any signs of agitation. From then on B attended therapy sessions in a wheel chair. This seemed to prevent B’s agitation from the outset of the sessions and allowed the therapy to enhance B’s brighter spirit.

### Acceptability

During the recruitment process, residents’ next of kin expressed positive attitudes to residents’ involvement in the study. Participant consent was provided for all residents.

On average, residents attended 75.11 % of available sessions. A mean number of 5 sessions were missed (standard deviation = 3.94). Reasons for missed sessions included resident illness, therapist illness, resident asleep, and resident choosing not to attend. On the part of the resident, the most common reason for lack of attendance was choosing not to attend (11 occurrences). These occurrences were contributable to only two residents, one of whom declined 9 times. Allowing for flexible start times of sessions was key to maximising attendance. It was commonly found that if a resident initially chose not to attend, they were happier to when approached again later.

Of the 6 completers receiving music therapy, 4 were accepting of the physiological sensors, one was not comfortable wearing either device, and one varied in her acceptance of it depending on her mood and presentation of symptoms. The Q sensor was generally more readily accepted due to ease of attachment and minimal personal ‘intrusion’. It is recommended that future studies should employ devices which require minimal input to attach, such as those worn on the wrist.

Regarding staff attitudes to the project, the results from the semi-structured interviews indicated a high level of acceptability of the intervention, and the study as a whole, from staff participants. No staff in the intervention group dropped out, in comparison to one carer in the control group who received no intervention. This was due to personal reasons and not study demands.

100 % intervention group staff reported they would recommend music therapy for someone with dementia, and all reported positive effects on residents. Comments included “I would recommend it for everyone”, and “please come back! Carry on”.

Staff talked of having enjoyed the presentations, and wanting to share what they had learnt with colleagues:

“They were quite beneficial to us”

“Amazing! I really could not believe… I actually wanted to go out the door and just go and tell everybody”.

Despite positive attitudes, it was not always possible to conduct the presentations due to staff shortages, and carers’ resulting shortage of time. On these occasions, presentations were prepared but given the following week.

### Preliminary outcomes

#### Effects of music therapy on symptoms and well being

**Fig. 2:**
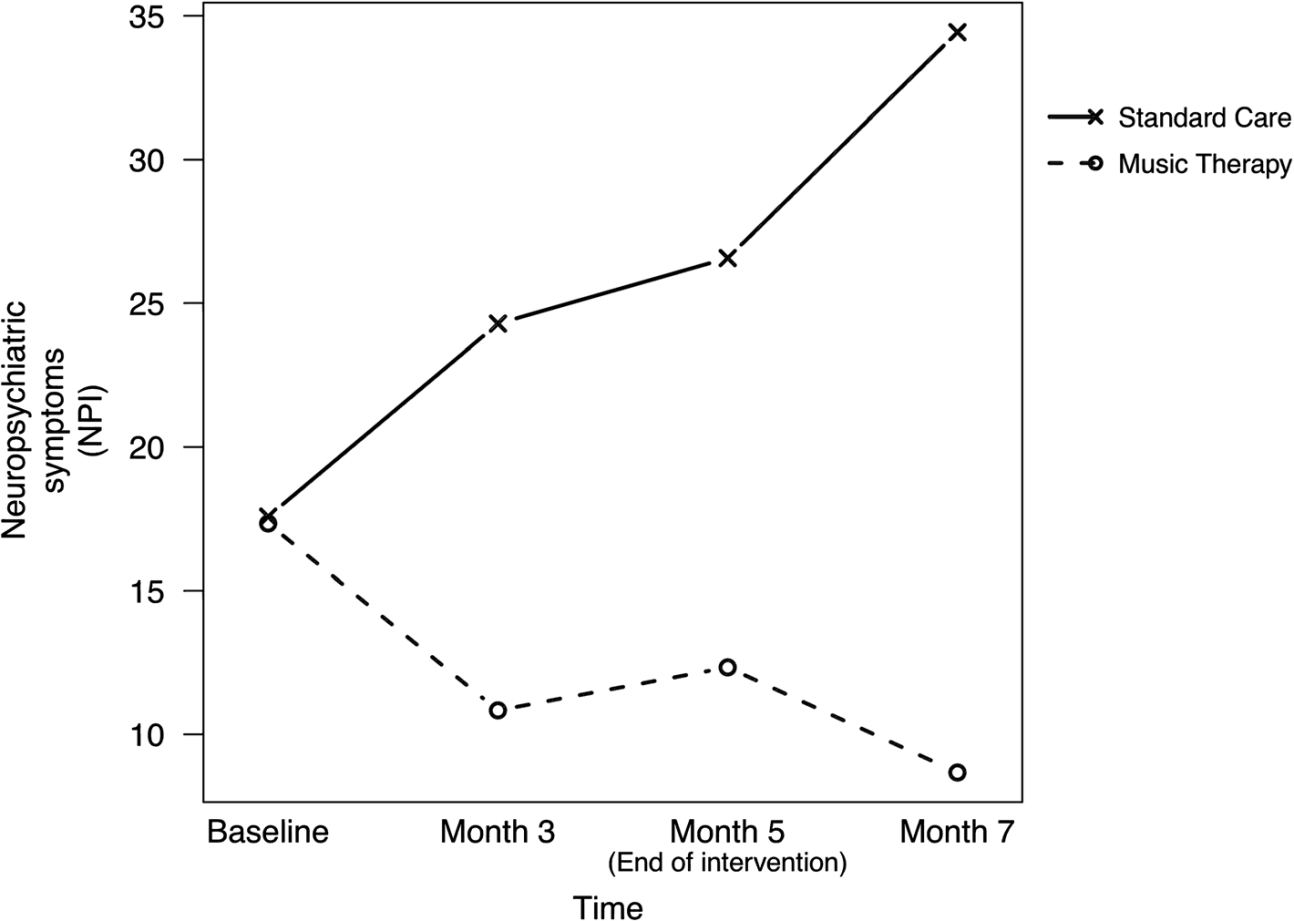
Mean scores for symptoms of dementia for music therapy and standard care groups

**Fig. 3:**
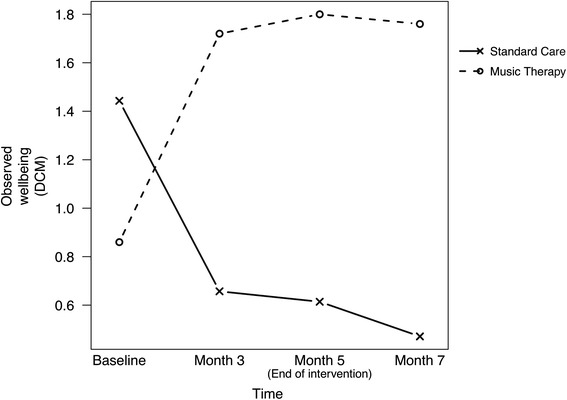
Mean scores for wellbeing levels for music therapy and standard care groups

The primary outcomes for symptoms of dementia and well-being are reported in Table 2. Mean Neuropsychiatric Inventory (NPI-Nursing Home Version) scores and Dementia Care Mapping (DCM) scores are displayed for the two groups. Figures 2 and 3 illustrate these in graphical form.

**Table 2:** Group means and percentage of Personal Enhancers during music therapy and standard care

Outcomes	Baseline		3 months		5 months		7 months	
	N	Mean (SD)	N	Mean (SD)	N	Mean (SD)	N	Mean (SD)
Symptom score (NPI-NH)								
Music therapy	6	17.33 (10.78)	6	10.83 (14.11)	6	12.33 (11.20)	6	8.67 (9.54)
Standard care	7	17.57 (6.08)	7	24.29 (8.86)	7	26.57 (7.14)	7	34.43 (7.37)
Disruptiveness score (NPI-NH)								
Music therapy	6	2.67 (4.68)	6	4.00 (5.62)	6	3.00 (4.38)	6	0.83 (1.33)
Standard care	7	3.00 (3.22)	7	10.71 (4.82)	7	10.71 (6.02)	7	13.86 (4.94)
Wellbeing score (DCM)								
Music therapy	5	0.86 (0.43)	5	1.72 (0.61)	5	1.80 (0.59)	5	1.76 (0.48)
Standard care	7	1.44 (0.49)	7	0.66 (0.61)	7	0.61 (0.49)	7	0.47 (0.68)
Personal enhancers (DCM-PE)								
Music therapy, %		87.50 (17.68)		61.90 (53.88)		74.90 (31.82)		71.80 (19.66)
Standard care, %		94.45 (7.85)		71.90 (39.75)		50.90 (43.70)		72.50 (20.08)

Table 3 summarises the contrast in changes between each pair of time points (Month 3-Baseline, Month 5-Baseline and Month 7-Baseline) between the two groups. Whilst dementia symptoms (NPI scores) in the standard care group increased over 5 months, dementia symptoms in the music therapy group decreased. This trend continued for both groups after the intervention ended (See Table 2 and Fig. 2). Significant differences between groups were found for each pair of time points. The greatest change between groups was found between baseline and Month 7 (−25.52; 95 % CI: [−39.10 to −11.95; *p* = 0.002]). This corresponded to a large effect size (2.32).

**Table 3:** Changes in music therapy versus standard care

Outcome	Month 3-Baseline						Month 5-Baseline						Month 7-Baseline					
	*n*	Mean difference	95 % confidence interval	Standard error	Effect size *d*	*p*	*n*	Mean difference	95 % confidence interval	Standard error	Effect size *d*	*p*	*n*	Mean difference	95 % confidence interval	Standard error	Effect size *d*	*p*
Symptoms (NPI-NH)																		
Standard Care	7	−13.21	−24.50 to −1.93	5.13	1.44	0.026	7	−14.00	−24.25 to −3.76	4.66	1.69	0.012	7	−25.52	−39.10 to −11.95	6.17	2.32	0.002
Music Therapy	6						6						6					
Disruptiveness (NPI-NH)																		
Standard care	7	−6.38	−11.53 to −1.23	1.52	2.34	0.02	7	−7.38	−11.20 to −3.57	1.73	2.39	0.001	7	−12.69	−18.50 to −6.88	2.64	2.69	0.001
Music therapy	6						6						6					
Wellbeing (DCM)																		
Standard care	7	1.65	0.71 to 2.58	0.42	2.28	0.003	7	4.14	1.97 to 6.31	0.97	2.48	0.002	7	1.87	1.24 to 2.50	0.28	3.85	<0.001
Music therapy	5						5						5					
Personal enhancers (DCM)																		
Standard care		−24.08	−97.47 to 49.32	17.06		0.294		−28.08	−86.70 to 30.55	13.63		0.176		−18.83	−37.68 to 0.026	4.38		0.050
Music therapy																		

The results for the first three rows above have been obtained using the SPSS repeated measures analysis of variance procedure. The Greenhouse-Geisser statistic for checking the correlation structure is 0.85 for Symptom (NPI-NH), 0.78 for Disruptiveness (NPI-NH), and 0.82 for Wellbeing (DCM), and this has led to the decision to use the SPSS results assuming “sphericity”

**Fig. 4:**
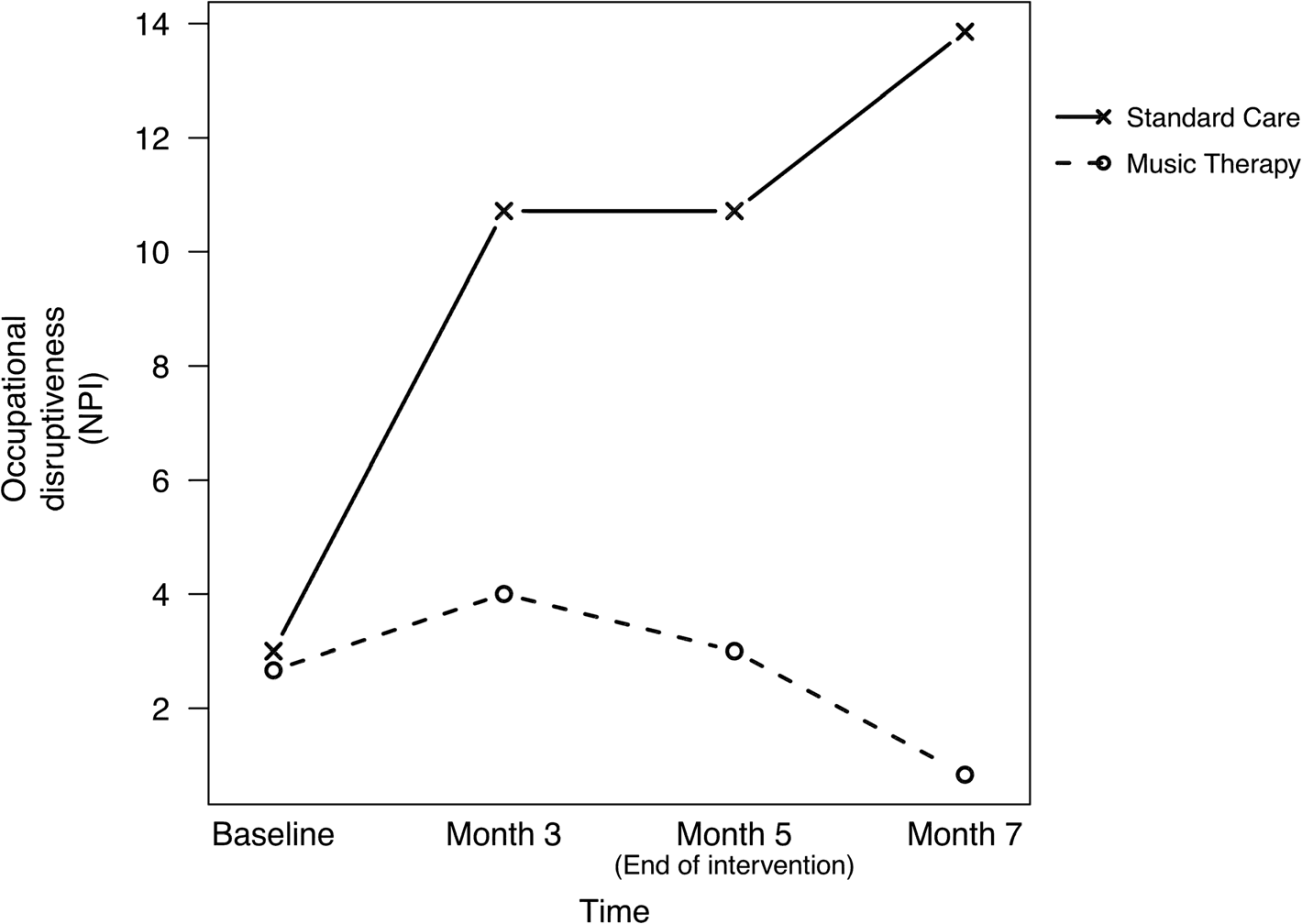
Mean scores for occupational disruptiveness for music therapy and standard care group

Staff perceptions of residents’ levels of occupational disruptiveness were also assessed. Staff were asked how highly they felt each residents’ symptoms were disruptive to their daily delivery of care. This is illustrated graphically in Fig. 4.

Occupational disruptiveness in the standard care group was found to increase steadily over the course of the project, and continued to increase during the 2 months following the cessation of the music therapy programme (an overall increase of 10.86). Within the music therapy group, an increase in residents’ occupational disruptiveness was also observed from Baseline to Month 3 (1.33). This was then followed by a steady decrease from Month 3 to Month 7 (−3.17). The greatest difference between groups was found between baseline and month 7 (−12.69, 95 % CI: [−18.50 to −6.88; *p* = 0.001]) with an effect size of 2.69.

In terms of wellbeing (DCM scores), there was an increase over time in the music therapy group from baseline to month 7. In contrast, the wellbeing level for the standard care group decreased over this time period (See Table 2 and Fig. 3). All differences between groups were statistically significant for each pair of time points with the greatest change found between baseline and month 5 (4.14, 95 % CI: [1.97 to 6.31; *p* = 0.002]) with a large effect size of 2.48. Both groups displayed a decrease in wellbeing between month 5 and month 7 when there was no intervention (See Table 2 and Fig. 3).

Overall the analysis of variance indicated significant differences between the standard care and music therapy groups for the levels of symptoms (13.42, 95 % CI: [4.78 to 22.07; *p* = 0.006]), occupational disruptiveness (6.95, 95 % CI: [2.43 to 11.47; *p* = 0.006]) and wellbeing (−0.74, 95 % CI: [−1.15 to −0.33; *p* = 0.003]).

#### Effects of music therapy on caregiving

During the Dementia Care Mapping to observe the interactions between staff and resident participants, occurrences of personally enhancing interactions were identified and coded. Table 3 reports the patterns of change on the levels of Personal Enhancers in both groups. However, differences between the groups were not statistically significant (−1.59; 95 % CI: [−127.81, 124.64; p = 0.962]). The impact of music therapy on caregiving, however, was further explored qualitatively with the analysis of carer participants’ semi-structured interviews at the end of the intervention.

#### Semi-structured interview summary

The semi-structured interviews provided insight into carers’ perceptions of the music therapy programme and the video presentations. Seven carers in the music therapy group were interviewed, and asked whether they felt the intervention had had an impact on the residents or their own caregiving.

#### Effect on residents

Carers reported beneficial effects of the intervention on residents, in particular on mood and emotion and sensorimotor functioning, as well as on self-expression and communication, memory, agitation, apathy, anxiety and aberrant motor behaviour. One carer stated, ‘It’s lightened her mood and it brings back memories for her about her husband, and she talks about it afterwards sometimes as well.’

#### Effect on carers and their daily practice

Carers also reported a positive impact of the music therapy presentations on themselves and their own work. Most commonly, they talked of gaining increased insight into residents, i.e. personal history, symptom causes and cognitive functioning. Other effects included enhanced interaction techniques, altered mood and enhanced communication and relationship with residents:

A carer noted that ‘different residents like particular songs, or maybe a particular, like, pace of song. Maybe a more slower song, especially before personal care if they’re agitated, a more slower song sang nice and softly… it’s made me notice that a different impact … So, if they’re agitated before personal care, a nice slow song. But then, once they’re in a good mood, they’re quite- and they’re up for a nice fast song and clap or dance around the room, do you know what I mean? It just varies. It all varies.’

#### Attitudes towards future training opportunities

The study also explored whether carers felt they would benefit from training workshops on music therapy techniques. Carers expressed an interest in learning specific musical skills, songs and instruments. They also talked of wanting to increase their self-confidence in using music with residents, and discussed the importance of gaining insight into residents’ cognitive and psychological needs:

A carer reflected she would like to ‘learn more about being aware of what to look out for… for example, Elsie being quite sensitive to other noises in the background. Cause I, personally learnt – communication-wise- a lot better with Elsie since the whole music therapy… learn to look for certain signs, or, however you’ve been doing it.’ [*Name changed to protect identity*]

### Harms

No adverse effects were observed as a result of the trial.

## Discussion

### Findings

The intent of our study was to explore the feasibility of carrying out this 5-month music therapy programme in dementia residential homes. By involving both care home residents and their carers as participants, we were able to explore the possible effects of the intervention on the residents’ neuropsychiatric symptoms of dementia and wellbeing. Additionally, we explored whether and how the intervention might bring changes to carers' caregiving. The music therapy programme appeared to show overall acceptability and validity. Although the completion rate in the intervention group was lower (67 %), we believe this was largely due to factors which were not related to the intervention itself, i.e. residents’ ill-health and carer’s personal reasons. The care staff participants were enthusiastic about the input of music therapy throughout the five months. They also displayed willingness and consistency in being involved in the interviews for Neuropsychiatric Inventory (NPI) and the observations for Dementia Care Mapping (DCM).

The study suggests beneficial effects of the music therapy programme on the symptoms of dementia and occupational disruptiveness as measured by the NPI. In addition, the music therapy programme was also associated with higher levels of well-being, as measured and indicated by the DCM. These findings concur with two earlier studies using NPI as the outcome measure to indicate the ameliorating effects of music therapy on neuropsychiatric symptoms of dementia [35, 37]. However, the two studies administered a 30 min music therapy session at a higher frequency (biweekly and 3 times a week), and in a group setting rather than 1:1. Therefore, it is difficult to draw conclusive comparisons as the intervention in our study was active individual music therapy administered once a week. Another study [52] reported possible benefits of biweekly active individual music therapy over a 6 week period in managing agitation disruptiveness in dementia care. This is supported by our findings, indicating that administering a lower dose of individual music therapy over a longer period could also potentially generate similar effects.

Regarding caregiving (measured by DCM), despite the promising effects reported in the semi-structured interviews, no statistical significance was detected. This outcome might have resulted from the limited number of observations (2 h observations using DCM were carried out at each of the 4 time points during the study). In future, more observations should be carried out. Pairing up carers and residents for observation should also be implemented in order to closely monitor changes in carers’ interaction with residents. Despite this shortcoming, the data from the semi-structured interviews suggested further acceptability and clinical utility of the music therapy programme.

This feasibility study is the first published music therapy study in which the intervention included post-session video presentations to carers in addition to weekly music therapy sessions to care home residents with dementia. The qualitative data revealed that the video presentations allowed the care staff to witness an array of music therapist-resident interactions in the sessions. These interactions appeared to be surprising and uplifting to the staff, and demonstrated how residents’ symptoms were reduced and how their remaining cognitive functions were activated. As a result, staff were motivated to utilise the learned insights and ideas from the video presentations in symptom management. During the trial, carers were able to report regularly what they had observed and accomplished in their interactions with the residents during their day-to-day care practice. This corresponds to the findings of Lawrence *et al.* [53] that providing staff with practice opportunities and embedding psychosocial interventions into daily care are necessary for successful implementation of such interventions. Moreover, this supports the use of videos as an effective teaching method for staff training [76] and suggests an educational role of the music therapist enhancing the multidisciplinary care planning and delivery. Significantly, our findings echo the importance of staff education and training which may be the most effective method in managing symptoms of dementia [23], and music therapists can play a valuable role in this context. Music therapists’ continuing post-qualification training, particularly the theoretical understanding of brain functions may also be a supporting factor for allowing music therapists to explain clients’ health and provide practical methods of symptom management, taking neuroscientific research findings into account.

### Limitations

There are challenges in conducting trials of psychosocial interventions in dementia long-term care settings [64]. We believe that the limitations of this trial are mainly due to it being undertaken on a smaller scale, due to being a feasibility study. The small sample size and resulting insufficient data led to the exclusion of the factor Home in the statistical analysis. This limits the test power to detect between-group differences despite the statistical significance in some of our results. Furthermore, due to the exploratory nature of the feasibility study, no multiple outcomes or sample size calculation were performed. Therefore, the results should be interpreted with a level of caution. In addition, the large effect sizes (>0.8) are also difficult for interpretations of any clinical significance. Another constraining factor to the sample size was the availability of the day of the week we were able to carry out the intervention. During the trial, one home received one day per week of music therapy input and the other home received two. This restricted the number of residents receiving music therapy, considering the amount of time required for the music therapists to carry out the therapy sessions, review video recordings and present to the staff on the same day. This consideration was also taken into account during the recruitment process and contributed to the low recruitment rate for the resident participants (22 %, 17 out of 75 assessed for eligibility). For future studies, more music therapy days per week and increased staffing for conducting observations should be sought for each participating home. This, along with modified inclusion criteria, will allow a higher recruitment rate for resident participants.

Issues with the randomisation were also encountered. The randomisation was administered to units within the homes. This could have caused contamination across the control and intervention groups. Although staff participants were advised to only work within the unit they were randomised to, there was no guarantee of this due to occasional staff shortages. In a follow up study, cluster randomisation would need to be administered to homes instead of units. Video presentations to the staff in the intervention group were thought to be the key element in detecting the difference of symptom management between intervention and control group. However, some degrees of the Hawthorne effect [77] could have been generated when the staff who received the presentations also provided outcomes for the NPI. This should be rectified in a follow up study by recruiting another group of staff in addition to the intervention and control groups. This staff group who also work with the residents should be blinded and provide outcomes without receiving any intervention.

As previously stated, the number and length of the DCM observations were limited due to the availability of time, days and observer in the week for data collection. This might have not captured an accurate representation of the course of residents’ wellbeing over time. More flexibility in scheduling for intervention and data collection should be considered in the design of a follow up study.

## Conclusions

This feasibility study addressed the process of the development and delivery of a music therapy programme that incorporated the involvement of care staff in managing neuropsychiatric symptoms of dementia for care home residents. The programme highlighted the importance of music therapist-carer communication in order to maximise the effects of weekly active music therapy on the residents’ care, wellbeing and symptom management. Nonetheless, it identified the role of music therapists not only in delivering the sessions but also in providing knowledge and skills that are clinically relevant to care delivery. The results of the study suggested potential sustained benefits on residents’ wellbeing and symptoms over the 5 month period of intervention and two month period post-intervention. With modifications in the study design, the protocol warrants a more rigorous evaluation in a larger RCT. This research is at an early stage of development. However, exploring the feasibility and challenges in the work provides a picture that approximates the reality of day-to-day life and care in dementia care homes. This is useful in planning and delivering broader innovative therapeutic inputs to improve the wellbeing of people with dementia in care homes. The future research should endeavour to satisfy similar ecological validity. The working mechanisms of music therapy sessions still await scientific investigation. The use of psychophysiological measures may illuminate the agents of change acting on residents’ internal states within sessions. This will help further clarify the clinical utility of music therapy in dementia care.

## Abbreviations

NPI: Neuropsychiatric inventory
DCM: Dementia care mapping
GDS: Global deterioration scale

## References

[1] Alzheimer’s Disease International (ADI). World Alzheimer report: Journey of Caring: An analysis of long-term care for dementia. 2013. http://www.alz.co.uk/research/WorldAlzheimerReport2013.pdf. Accessed 1 April 2015.

[2] Margallo-Lana M, Swann A, O’Brien J, Fairburn A, Reichelt K, Potkins D, Ballard C (2001). Prevalence and pharmacological management of behavioural and psychological symptoms amongst dementia sufferers living in care environments. Int J Geriatr Psychiatry.

[3] Zuidema S, Koopmans R, Verhey F (2007). Prevalence and predictors of neuropsychiatric symptoms in cognitively impaired nursing home patients. J Geriatr Psychiatry Neurol.

[4] Aalten P, de Vugt ME, Jaspers N, Jolles J, Verhey FRJ (2005). The course of neuropsychiatric symptoms in dementia. Part I: findings from the two-year longitudinal Maasbed study. Int J Geriatr Psychiatry.

[5] Douglas S, James I, Ballard C (2004). Non-pharmacological interventions in dementia. Adv Psychiatr Treat.

[6] Kitwood T (1997). Dementia Reconsidered: The Person Comes First.

[7] Kitwood T, Bredin K (1992). Towards a theory of dementia care: personhood and well-being. Ageing Soc.

[8] Bains J, Birks JS, Dening TR (2002). Antidepressants for treating depression in dementia. Cochrane Database Syst Rev.

[9] Lonergan E, Luxenberg J, Colford J (2002). Haloperidol for agitation in dementia. Cochrane Database Syst Rev.

[10] Cooper C, Mukadam N, Katona C, Lyketsos CG, Blazer D, Ames D, Rabins P, Brodaty H, de Mendonca LC, Livingston G (2013). Systematic review of the effectiveness of pharmacologic interventions to improve quality of life and well-being in people with dementia. Am J Geriatr Psychiatry.

[11] Prudent M, Dramé M, Jolly D, Trenque T, Parjoie R, Mahmoudi R, Lang PO, Somme D, Boyer F, Lanièce I, Gauvain JB, Blancahrd F, Novella JL (2008). Potentially inappropriate use of psychotropic medications in hospitalized elderly patients in France: cross-sectional analysis of the prospective, multicentre SAFEs cohort. Drugs Aging.

[12] Gustafsson M, Karlsson S, Gustafson Y, Loevheim H (2013). Psychotropic drug use among people with dementia – a six-month follow-up study. BMC Pharmacol Toxicol.

[13] Department of Health. ‘Living well with dementia, A National Dementia Strategy’. The National Dementia strategy in England. Br J Med Med Res. 2009;338. [http://dx.doi.org/10.1136/bmj.b931]

[14] Department of Health (2009). The Use of Antipsychotic Medication for People with Dementia: Time for Action.

[15] Odell-Miller H, Sandford S. Update on Music Therapy in the United Kingdom. Voices Resources. 2009. http://testvoices.uib.no/community/?q=country/monthuk_march2009. Accessed 1 April 2015.

[16] World Federation of Music Therapy. Announcing WFMT’s NEW Definition of Music Therapy. 2011. http://www.wfmt.info/2011/05/01/announcing-wfmts-new-definition-of-music-therapy/. Accessed 1 April 2015.

[17] Health Care Professions Council. Standards of Proficiency: Arts Therapists. 2013. http://www.hpc-uk.org/assets/documents/100004FBStandards_of_Proficiency_Arts_Therapists.pdf. Accessed 1 April 2015.

[18] Health Care Professions Council. Standards of Education and Training. 2014. http://www.hpc-uk.org/assets/documents/10000BCF46345Educ-Train-SOPA5_v2.pdf. Accessed 1 April 2015.

[19] National Institute for Health and Care Excellence. NICE guidelines 42: Dementia. 2006. http://pathways.nice.org.uk/pathways/dementia. Accessed 1 April 2015.

[20] Brotons M, Aldridge D (2000). An Overview of the Music Therapy Literature Relating to Elderly People. Music Therapy in Dementia Care.

[21] Brotons M, Pickett-Cooper P (1996). The effects of music therapy intervention on agitation behaviors of Alzheimer’s disease patients. J Music Ther.

[22] Koger SM, Chapin K, Brotons M (1999). Is music therapy an effective intervention for dementia? A meta-analytic review of literature. J Music Ther.

[23] Livingston G, Johnston K, Katona C, Paton J, Lyketsos CG (2005). Systematic review of psychological approaches to the management of neuropsychiatric symptoms of dementia. Am J Psychiatry.

[24] McDermott O, Crellin N, Ridder HM, Orrell M (2012). Music therapy in dementia: a narrative synthesis systematic review. Int J Geriatr Psychiatry.

[25] Raglio A, Bellelli G, Mazzola P, Bellandi D, Giovagnoli AR, Farina E, Trabucchi M (2012). Music, music therapy and dementia: a review of literature and the recommendations of the Italian Psychogeriatric Association. Maturitas.

[26] Ueda T, Suzukamo Y, Sato M, Izumi S-I (2013). Effects of music therapy on behavioural and psychological symptoms of dementia: a systematic review and meta-analysis. Ageing Res Rev.

[27] Guetin S, Charras K, Berard A, Arbus C, Berthelon P, Blanc F, Blayac JP, Bonte F, Bouceffa JP, Clement S, Ducourneau G, Gzil F, Laeng N, Lecourt E, Ledoux S, Platel H, Thomas-Anterion C, Touchon J, Vrait FX, Leger J-M (2013). An overview of the use of music therapy in the context of Alzheimer’s disease: a report of a French expert group. Dementia.

[28] Lord T, Garner E (1993). Effects of music on Alzheimer patients. Percept Mot Skills.

[29] Cohen-Mansfield J, Werner P (1997). Management of verbally disruptive behaviors in nursing home residents. J Gerontol A Biol Sci Med Sci.

[30] Denney A (1997). Quiet music. An intervention for mealtime agitation?. J Gerontol Nurs.

[31] Ragneskog H, Kihlgren M, Karlsson I, Norberg A (1996). Dinner music for demented Patients analysis of video-recorded observations. Clin Nurs Res.

[32] Remington R (2002). Calming music and hand massage with agitated elderly. Nurs Res.

[33] Gerdner L (2000). Effects of individualized versus classical “relaxation” music on the frequency of agitation in elderly persons with Alzheimer’s disease and related disorders. Int Psychogeriatr.

[34] Clark ME, Lipe AW, Bilbrey M (1998). Use of music to decrease aggressive behaviors in people with dementia. J Gerontol Nurs.

[35] Sung H, Chang S, Lee W, Lee M (2006). The effects of group music with movement intervention on agitated behaviours of institutionalized elders with dementia in Taiwan. Complement Ther Med.

[36] Svansdottir HB, Snaedal J (2006). Music therapy in moderate and severe dementia of Alzheimer’s type: a case–control study. Int Psychogeriatr.

[37] Raglio A, Bellelli G, Traficante D, Gianotti M, Ubezio MC, Villani D, Trabucchi M (2008). Efficacy of music therapy in the treatment of behavioral and psychiatric symptoms of dementia. Alzheimer Dis Assoc Disord.

[38] Raglio A, Oasi O, Gianotti M, Manzoni V, Bolis S, Ubezio MC, Gentile S, Villani D, Stramba-Badiale M (2010). Effects of music therapy on psychological symptoms and heart rate variability in patients with dementia. A pilot study. Curr Aging Sci.

[39] Raglio A, Bellelli G, Traficante D, Gianotti M, Ubezio M, Gentile S, Villani D, Trabucchi M (2010). Efficacy of music therapy treatment based on cycles of sessions: a randomised controlled trial. Aging Ment Health.

[40] Ledger A, Baker F (2007). An investigation of long-term effects of group music therapy on agitation levels of people with Alzheimer’s disease. Aging Ment Health.

[41] Clair A, Ebberts A (1997). The effects of music therapy on interactions between family caregivers and their care receivers with late stage dementia. J Music Ther.

[42] Kumar AM, Tims F, Cruess DG, Mintzer MJ, Ironson G, Loewenstein D, Cattan R, Fernandez JB, Eisdorfer C, Kumar M (1999). Music therapy increases serum melatonin levels in patients with Alzheimer’s disease. Altern Ther Health Med.

[43] Okada K, Kurita A, Takase B, Otsuka T, Kodani E, Kusama Y, Atarashi H, Mizuno K (2009). Effects of music therapy on autonomic nervous system activity, incidence of heart failure events, and plasma cytokine and catecholamine levels in elderly patients with cerebrovascular disease and dementia. Int Heart J.

[44] Suzuki M, Kanamori M, Watanabe M, Nagasawa S, Kojima E, Ooshiro H, Nakahara D (2004). Behavioral and endocrinological evaluation of music therapy for elderly patients with dementia. Nurs Health Sci.

[45] Takahashi T, Matsushita H (2006). Long-term effects of music therapy on elderly with moderate/severe dementia. J Music Ther.

[46] Ashida S (2000). The effect of reminiscence music therapy sessions on changes in depressive symptoms in elderly persons with dementia. J Music Ther.

[47] Ridder HM, Wigram T, Ottesen AM (2009). A pilot study on the effects of music therapy on frontotemporal dementia—developing a research protocol. Nordic J Music Ther.

[48] Vink AC, Zuidersma M, Boersma F, Jonge P, Zuidema SU, Slaets JPJ (2013). The effect of music therapy compared with general recreational activities in reducing agitation in people with dementia: a randomised controlled trial. Int J Geriatr Psychiatry.

[49] Schall A, Haberstroh J, Pantel J. Time series analysis of individual music therapy in dementia. GeroPsych. 2015; [http://dx.doi.org/10.1024/1662-9647/a000123] Accessed 14 July 2015.

[50] Solé C, Mercadal-Brotons M, Galati A, De Castro M (2014). Effects of group music therapy on quality of life, affect, and participation in people with varying levels of dementia. J Music Ther.

[51] Vink AC, Bruinsma MS, Scholten RJPM (2003). Music therapy for people with dementia. Cochrane Database Syst Rev.

[52] Ridder HMO, Stige B, Qvale LG, Gold C (2013). Individual music therapy for agitation in dementia: an exploratory randomized controlled trial. Aging Ment Health.

[53] Lawrence V, Fossey J, Ballard C, Moniz-Cook E, Murray J (2012). Improving quality of life for people with dementia in care homes: making psychosocial interventions work. Br J Psychiatry.

[54] Craig P, Dieppe P, Macintyre S, Michie S, Nazareth I, Petticrew M. Developing and evaluating complex interventions: the new Medical Research Council guidance. BMJ. 2008;337. 10.1136/bmj.a1655 PMC2769032 18824488

[55] Campbell M, Fitzpatrick R, Haines A, Kinmonth AL, Sandercock P, Spiegelhalter D, Tyrer P (2000). Framework for the design and evaluation of complex interventions to improve health. BMJ.

[56] Reisberg B, Ferris SH, de Leon MJ, Crook T (1982). The Global Deterioration Scale for assessment of primary degenerative dementia. Am J Psychiatry.

[57] Odell-Miller H, Wigram T, Saperston B, West R (1995). Approaches to Music in Psychiatry with Specific Emphasis upon a Research Project with the Elderly Mentally Ill. The Art and Science of Music Therapy: A Handbook.

[58] Panksepp J, Strongman K (1991). Affective Neuroscience: A Conceptual Framework for the Neurobiological Study of Emotions. International Reviews of Emotion Research.

[59] Sander D, Armony J, Vuilleumier P (2013). Models of Emotion: The Affective Neuroscience Approach. The Cambridge Handbook of Human Affective Neuroscience.

[60] Koelsch S, Armony J, Vuilleumier P (2013). Emotion and Music. The Cambridge Handbook of Human Affective Neuroscience.

[61] Brueck C, Kreifelts B, Ethofer T, Wildgruber D, Armony J, Vuilleumier P (2013). Emotional Voices: The Tone of (true) Feelings. The Cambridge Handbook of Human Affective Neuroscience.

[62] Atkinson AP, Armony J, Vuilleumier P (2013). Bodily Expressions of Emotion: Visual Cues and Neural Mechanisms. The Cambridge Handbook of Human Affective Neuroscience.

[63] George N, Armony J, Vuilleumier P (2013). The Facial Expressions of Emotions. The Cambridge Handbook of Human Affective Neuroscience.

[64] Vernooij-Dassen M, Vasse E, Zuidema S, Cohen-Mansfield J, Moyle W (2010). Psychosocial interventions for dementia patients in long-term care. Int Psychogeriatr.

[65] Dugmore O, Orrell M, Spector A. Qualitative studies of psychosocial interventions for dementia: a systematic review. Aging Ment Health*.* 2015. [DOI: 10.1080/13607863.2015.1011079]. 10.1080/13607863.2015.1011079 25748797

[66] Wood S, Cummings JL, Hsu MA, Barclay T, Wheatley MV, Yarema KT, Schnelle JF (2000). The use of the Neuropsychiatric Inventory in nursing home residents, characterization and measurement. Am J Geriatr Psychiatry.

[67] Aalten P, Verhey FR, Boziki M, Bullock R, Byrne EJ, Camus V, Robert PH (2007). Neuropsychiatric syndromes in dementia. Results from the European Alzheimer Disease Consortium: part I. Dement Geriatr Cogn Disord.

[68] Ballard C, O’Brien JT, Reichelt K, Perry E (2002). Aromatherapy as a safe and effective treatment for the management of agitation in severe dementia: the results of a double blind, placebo-controlled trial with Melissa. J Clin Psychiatry.

[69] Brooker D, Surr C (2006). Dementia-care mapping (DCM): initial validation of DCM 8 in UK field trials. Int J Geriatr Psychiatry.

[70] Brooker D, Duce L (2000). Well-being and activity in dementia: a comparison of group reminiscence therapy, structured goal-directed group activity, and unstructured time. Aging Ment Health.

[71] Gigliotti CM, Jarrott SE, Yorgason J (2004). Harvesting health: effects of three types of horticultural therapy activities for persons with dementia. Dementia.

[72] DeCoster J. Spreadsheet for converting effect size measures. 2012. http://www.stat-help.com/spreadsheets/Converting%20effect%20sizes%202012-06-19.xls. Accessed 1 April 2015.

[73] Ochsner KN, Gross JJ (2005). The cognitive control of emotion. Trends Cogn Sci.

[74] Damasio AR (1994). Descartes’ error: Emotion, reason and the human brain.

[75] Damasio AR, Everitt BJ, Bishop D (1996). The somatic marker hypothesis and the possible functions of the prefrontal cortex [and discussion]. Philos Trans R Soc London [Biol].

[76] Kuske B, Hanns S, Luck T, Angermeyer MC, Behrens J, Riedel-Heller SG (2007). Nursing home staff training in dementia care: a systematic review of evaluated programs. Int Psychogeriatr.

[77] Landsberger HA (1958). Hawthorne Revisited.

